# Therapeutic drug monitoring in children and adolescents treated with quetiapine for different psychiatric disorders: results of the TDM-VIGIL pharmacovigilance study

**DOI:** 10.1007/s00702-026-03253-6

**Published:** 2026-08-17

**Authors:** Alina Kaiser, Karin Maria Egberts, Hans-Willi Clement, Marcel Romanos, Andreas Karwautz, Paul L. Plener, Christoph U. Correll, Uwe Malzahn, Peter Heuschmann, Stefan Unterecker, Maike Scherf-Clavel, Hans Rock, Tobias Banaschewski, Hartmut Imgart, Michael Kaess, Tobias Renner, Su-Yin Reuter-Dang, Gerd Schulte-Körne, Frank Theisen, Susanne Walitza, Christian Fleischhaker

**Affiliations:** 1 Department of Child and Adolescent Psychiatry and Psychotherapy, University Medical Center Freiburg, Freiburg, Germany; 2 Department of Child and Adolescent Psychiatry, Psychosomatics and Psychotherapy, Center for Mental Health, University Hospital of Wuerzburg, Wuerzburg, Germany; 3 Department of Child and Adolescent Psychiatry, Medical University Vienna, Vienna, Austria; 4 Department of Child and Adolescent Psychiatry, Charité Universitätsmedizin Berlin, Berlin, Germany; 5 Department of Psychiatry, The Zucker Hillside Hospital, Northwell Health, Glen Oaks, NY USA; 6 Department of Psychiatry and Molecular Medicine, Donald and Barbara Zucker School of Medicine at Hofstra/Northwell, Hempstead, NY USA; 7 Department of Child and Adolescent Psychiatry/Psychotherapy, University Hospital Ulm, Ulm, Germany; 8 Clinical Trial Center Wuerzburg, University Hospital Wuerzburg, Wuerzburg, Germany; 9 Institute of Clinical Epidemiology and Biometry, University of Wuerzburg, wuerzburg, Germany; 10 Department of Psychiatry, Psychosomatics and Psychotherapy, Center of Mental Health, University Hospital Wuerzburg, Wuerzburg, Germany; 11 Central Information Office, Department of Neurology, Philipps University of Marburg, Marburg, Germany; 12 Department of Child and Adolescent Psychiatry and Psychotherapy, Central Institute of Mental Health, Medical Faculty Mannheim, Heidelberg University, German Center for Mental Health (DZPG), partner site Mannheim-Heidelberg-Ulm, Mannheim, Germany; 13 Clinic for Psychosomatics and Psychotherapy, Parkland-Clinic, Academic Teaching Hospital for the University Gießen, Bad Wildungen, Germany; 14 Clinic for Child and Adolescent Psychiatry, Center for Psychosocial Medicine, University Hospital Heidelberg, Heidelberg, Germany; 15 University Hospital of Child and Adolescent Psychiatry and Psychotherapy, University of Bern, Bern, Switzerland; 16 Department of Child and Adolescent Psychiatry, Psychosomatics and Psychotherapy, University Hospital of Psychiatry and Psychotherapy Tuebingen, Center of Mental Health Tuebingen, Tuebingen, Germany; 17 Specialist practice and Medical Care Center for Child and Adolescent Psychiatry Munich, Munich, Germany; 18 Department of Child and Adolescent Psychiatry, Psychosomatics and Psychotherapy, Ludwig-Maximilians-University (LMU) Hospital, Munich, Germany; 19 Department of Child and Adolescent Psychiatry and Psychotherapy, Herz- Jesu-Krankenhaus gGmbH, Fulda, Germany; 20 Department of Child and Adolescent Psychiatry and Psychotherapy, Psychiatric University Hospital Zurich, University of Zurich, Zürich, Switzerland; 21 Zurich Center for Integrative Human Physiology, University of Zurich, Zürich, Switzerland; 22 Neuroscience Center Zurich, University of Zurich and ETH, Zurich, Switzerland

**Keywords:** Quetiapine, Children and adolescents, Schizophrenia, Serum concentration, Therapeutic drug monitoring, Pharmacovigilance

## Abstract

Although quetiapine is increasingly used off-label in children and adolescents with various psychiatric disorders, there is little evidence on the relationship between quetiapine dose and serum concentration, and therapeutic reference ranges (TRR) have not yet been established for this age group. The aim of this study was to evaluate whether the TRR described for adults is applicable to minors. In addition, the study aimed to investigate the relationship between the daily dose, serum concentration and clinical outcome. The clinical course of 77 patients (females = 78%) aged between 12 and 17 years treated with quetiapine in the multicentre pharmacovigilance study TDM-VIGIL was systematically followed, and serum concentrations were measured. A positive mean correlation was observed between the weight-adjusted daily dose and serum concentration (*r*_s_ = 0.580, *p* < 0.001). Most patients (84.7%) experienced no ADRs or ADRs not significantly interfering with the patients’ functioning. A positive linear relationship between the therapeutic response and serum concentration was found (*r*_s_ = 0.238, *p* = 0.006). Only 38.5% of all quetiapine serum concentrations were within the recommended TRR described for adults. Using two different calculation methods, mean ± SD and IQR, our data hinted at considerably lower TRR for minors of about 14–291 ng/ml and 53–234 ng/ml respectively, compared to the suggested TRR for adults. This study has the potential to contribute to the definition of a TRR for quetiapine in minors. However, further controlled studies with larger sample sizes are needed to verify the preliminary results.

**Supplementary Information:**

The online version contains supplementary material available at https://doi.org/10.1007/s00702-026-03253-6.

## Introduction

Therapeutic Drug Monitoring (TDM) is considered as an effective method for individual dosing of psychotropic drugs and for safety monitoring throughout pharmacotherapy (Schoretsanitis et al. 2020). Whilst TDM has become established as standard practice in adult psychiatry for many antipsychotic drugs, resulting in enhanced efficacy and reduced risks of adverse drug reactions (ADRs) (Hiemke et al. 2018), its potential advantages also apply to minors (Egberts et al. 2011) and other classes of drugs, e.g. SSRI (Strawn et al. 2021). In recent decades, various studies have provided initial evidence for optimising the efficacy (Hermans et al. 2024; Taurines et al. 2022), tolerability (Egberts et al. 2022) and costs (Fekete et al. 2023) of psychotropic drug use through TDM in minors. Furthermore, minors possibly particularly benefit from TDM due to their increased vulnerability for ADRs (Kloosterboer et al. 2020).

Quetiapine, a second-generation antipsychotic, acts as a low-affinity dopamine D_2_- and a moderate affinity serotonin 5HT_2A_ receptor antagonist (Richelson and Souder 2000). Additionally quetiapine acts as an antagonist at Alpha-1 and H1 receptors and as a partial agonist at 5-HT_1A_ receptors which supposedly contributes to its antidepressant effect (Jensen et al. 2008; Richelson and Souder 2000).

Quetiapine immediate release is usually administered twice-daily, whereas a once-daily extended-release formulation is also available, with both formulations showing similar plasma concentration-time curves when prescribed at the same total daily dose (Bui et al. 2013; Figueroa et al. 2009). The AGNP consensus guidelines for TDM suggest a therapeutic reference range (TRR) of 100–500 ng/ml for the treatment of schizophrenia in adults (Hart et al. 2026; Hiemke et al. 2018). At doses within this recommended TRR, quetiapine has a linear pharmacokinetic profile with an elimination half-time of approximately 7 h (DeVane and Nemeroff 2001), in which no apparent differences in pharmacokinetics have been observed in adolescents compared to adults (McConville et al. 2000). The main route of elimination is through hepatic metabolization mainly via CYP3A4 (DeVane and Nemeroff 2001).

Clinical studies have suggested good tolerability of quetiapine in minors and generally consistent safety profiles with the profiles of quetiapine reported in adults (Findling et al. 2012, 2013; Pathak et al. 2013). Frequently observed adverse events in children and adolescents include somnolence, sedation, dizziness, and headaches, similar to profiles reported in adults (Findling et al. 2013). The risk of weight gain, however, has been suggested to be higher in minors than in adults (Correll 2008). In the USA, the Food and Drug Administration has approved the use of quetiapine in children and adolescents for the treatment of schizophrenia from the age of 13 and for the treatment of bipolar disorder from the age of 10, whereas the European Medicines Agency has not approved the use of quetiapine in minors, as it recognized not enough data to support its use in this age group (Gerlach et al. 2014). As a result, the off-label use of quetiapine in children and adolescents is common.

The primary aim of this study was to evaluate, whether the TRR described for adults when using quetiapine is also applicable to minors and to define diagnosis-dependent therapeutic reference ranges specifically for children and adolescents treated with quetiapine. Secondly, the study sought to analyse the relationship between the daily dose, serum concentration, clinical outcome and possible modulating factors in children and adolescents. The relationship between the daily dose and the serum concentration was described using dose-related concentration factors. Unlike the TRR, which interprets TDM results based on pharmacodynamics, the dose-related reference range follows a pharmacokinetic approach. The concept of dose-related concentration factors was invented to help predict expected drug concentrations in patients. It is defined as a concentration range within which a drug concentration is expected according to pharmacokinetic studies and is calculated by multiplying the mean DRC-factors low and high, calculated from mean DRC ± standard deviation specified for a particular drug, by the daily dose (Fekete et al. 2020). The DRC-range is considered as the range expected in 68% of the values of a reference population (Hart et al. 2026; Hiemke et al. 2018).

## Methods

### Study design and study population

This study was part of the multicentre, large simple clinical trial (phase IIIb) ‘*TDM VIGIL’*,* subproject 1*,* EudraCT number 2013–004881-33*, funded by the German Federal Institute for Drugs and Medical Devices, Bonn (BfArM, number V-15322/68605/2013–2018). The overall aim of the TDM-VIGIL pharmacovigilance study, described in detail elsewhere (Egberts et al. 2022), was to systematically collect data on prescribing patterns, therapeutic response, and ADRs associated with antidepressants and antipsychotics in children and adolescents diagnosed with various psychiatric and behavioural disorders. 710 patients from 18 different hospitals throughout Germany, Austria, and Switzerland were prospectively followed between 2014 and 2018. For data privacy reasons, all clinics were members of the competence network for TDM in child and adolescent psychiatry (www.tdm-kjp.com, Mehler-Wex et al. 2009). Demographic and clinical data, as well as medication regimes, were collected using an internet-based patient registry. Patients aged 4 to 18 years who were newly treated with antipsychotics or antidepressants in on-label or off-label use were eligible for participation regardless of the diagnosis and the treatment setting (inpatient, day clinic or outpatient). Written informed consent was obtained from the patients’ legal guardians, and if possible, from the patients themselves (informed assent from the age of 14 years). Patients were excluded from the study if there were absolute contraindications to the drug or if they were taking part in other clinical studies at the same time. The study was approved by the ethics committee of the University Hospital of Wuerzburg (301/13), and by the local ethic committees of all participating centres.

In addition to the general criteria mentioned above, further inclusion criteria were applied to the quetiapine sample. At least a baseline and one additional visit with a documented medication episode with quetiapine were required for inclusion in the study. Depending on the course of treatment, patients could have a varying number of visits and of valid TDM measurements included in the analyses. With this, the number of patients and assessments varied depending on the assessment, so that patients or visits could be involved in a certain sub-analysis despite being excluded from another. This resulted in different numbers of patients for different sub-analyses. Reasons for the exclusion of patients or assessments involved unfulfilled steady-state conditions, intoxication, or questionable treatment adherence.

### Data collection

Prior to the treatment with quetiapine, all patients underwent a physical examination which included the assessment of vital signs, height and body weight, an electrocardiography and blood tests of hepatic and renal function. Clinical diagnoses were determined by psychiatrists according to the 10th revision of the International Classification of Diseases (ICD-10). Multiple patients had been assigned more than one diagnosis. Patient characteristics, co-morbidities and medication regimes, including concomitant medications, were documented at a baseline-assessment directly before starting the treatment with quetiapine and at every follow-up visit to enable a direct comparison before and during treatment with quetiapine. The severity of psychopathology was assessed at baseline using the Clinical Global Impression Scale (CGI-S) from 1 = not at all ill, 2 = borderline mentally ill, 3 = mildly ill, 4 = moderately ill, 5 = markedly ill, 6 = severely ill and 7 = among the most extremely ill patients.

The clinical response to quetiapine was measured at the time of blood withdrawal by the attending physician. Improvement of symptoms in comparison to the state at baseline was rated according to the Clinical Global Impression Scale (CGI-I) on a scale of 1 = very much improved, 2 = much improved, 3 = minimally improved, 4 = unchanged, 5 = minimally worse, 6 = much worse and 7 = very much worse (Guy, 1976). In addition, the clinical effectiveness of quetiapine was assessed in relation to the time of initiation of treatment on a scale of 1 = very good, 2 = moderate, 3 = weak, and 4 = no response, whereupon patients with a response score of 1 or 2 were defined as responders to the medication. Medication-related ADRs were assessed using the Udvalg for Kliniske Undersøgelser-Side Effect Rating Scale (Lingjaerde et al., 1987) according to the following categories 1 = none, 2 = side effect does not significantly interfere with patient functioning, 3 = side effect significantly interferes with patient functioning and 4 = side effect outweighs therapeutic effect. Unlike the usual use of the CGI-S and the CGI-I referring to the overall therapeutic effect of each patient, the clinical response and the adverse events were assessed separately for each medication so that the results in this study refer exclusively to quetiapine. To enable a descriptive assessment of symptoms improving or deteriorating throughout medication, the Pediatric Adverse Event Rating Scale (PAERS) was used, ranging from 1 = mild, 2 = moderate, 3 = severe to 4 = extremely severe (March et al., 2007). It was designed to assess and quantify the severity of 56 symptoms on a five-point Likert scale with documentation being made regarding whether the symptoms were possibly related to the use of quetiapine.

Since the study was conducted on children and adolescents, the absolute weight gain provides only limited information about potential drug-related side effects, as growth and pubertal development significantly influence body weight at this age. Therefore, all weight values in this study were converted into age- and gender-specific percentiles and all valid BMI-values in our study measured at baseline and at the last assessment were converted into percentiles based on normal distribution using the coefficient of variation, the skewness and the median determined according to the reference percentiles for anthropometric measures based on the German Health Interview and Examination Survey for children and adolescents (Neuhauser et al. 2013).

Quetiapine serum concentrations were measured according to the consensus guidelines for therapeutic drug monitoring of the Society for Neuropsychopharmacology and Pharmacopsychiatry (Arbeitsgemeinschaft für Neuropsychopharmakologie und Pharmakopsychiatrie) (Hart et al. 2026; Hiemke et al. 2018). Blood was collected at steady-state and trough levels by withdrawing 7,5 ml from the cubital vein in the morning before the first daily dose of quetiapine and at least 12 h after the last drug administration if, quetiapine was taken twice daily. All serum concentrations were determined immediately (samples from Wuerzburg) or after postage to the TDM laboratory of the University Hospital Wuerzburg using an isocratic high-performance liquid chromatography method (Agilent 1200 series, Agilent Technologies Inc., Santa Clara, CA, USA) with UV-absorbance detection, as described in detail elsewhere (Albantakis et al. 2017; Gerlach et al. 2007). Each analytical series contained internal quality control samples and in addition, external control samples were assessed quarterly.

In this study, a preliminary DRC-factor low and high was calculated by dividing the mean drug concentration ± standard deviation by the mean daily dose. All assessments with valid data regarding the dose and the serum concentration were used to calculate the preliminary DRC-factor and the result was compared to the DRC-factor described in literature for 18–65 year old adults under normal conditions (Hart et al. 2026) and to DRC-factors described for minors (Fekete et al. 2020). The influence of all concomitant medications with *n* ≥ 5 patients (fluoxetine, escitalopram, sertraline, mirtazapine and aripiprazole) was examined.

As opposed to adults, no comparable therapeutic reference range has been described for children and adolescents treated with quetiapine. The consensus guidelines on therapeutic drug monitoring (Hiemke et al., 2011) proposed an approach on how to determine preliminary therapeutic reference ranges for medications for which a TRR has not yet been validated. According to this method, the mean serum concentration ± one standard deviation of patients who responded to drug therapy is considered the preliminary TRR. In addition, a second recommended method to define TRRs according to the 25th and 75th percentiles of serum concentrations measured in responding patients was used (Hart et al. 2021).

### Data analysis

Statistical analysis was performed using SPSS^®^ (version 28) and Microsoft Excel^®^ (version 15). Appropriate descriptive statistics are given according to the measurement scale of data and for interval and ratio scaled data, respectively, according to the properties of the observed distributions. All variables were tested for gaussian distributing using the Shapiro-Wilk test and additionally by visually analysing the corresponding Q-Q plots. For normally distributed variables, the Pearson coefficient (*r*_p_) was applied, whilst not normally distributed variables were tested using the Spearmann rank correlation coefficient (*r*_s_). Low correlation was defined as *r* < 0.2, moderate correlation as *r* = 0.2–0.5 and strong correlation as *r* > 0.5. Simple linear regression analysis was used to describe the relationship between the dose and the serum concentration and multiple linear regression was calculated to predict the serum concentration based on the dose, weight, gender, and the presence of concomitant medications. Differences between two groups were analysed using the nonparametric Mann-Whitney *U* test and for more than two independent samples by using the Kruskal-Wallis test. The Wilcoxon signed-ranks test for dependent samples was used to analyse the weight change after the treatment with quetiapine. The significance level was set at α = 0.05 (Table 1).

## Results

### Study population

The study population consisted of 77 patients (17 male, 60 female) with a mean age of 15.1 ± 1.1 (range 12 − 17) years. As most patients had undergone several visits, a total of 327 assessments was recorded. The average time interval between the first and the last assessment was 128.4 ± 107.1 (range 11 − 427) days. According to the CGI-S score, the mean severity of illness prior to treatment with quetiapine was 5.4 ± 0.8. Patients were treated with quetiapine for different psychiatric diagnoses, including affective disorders, accounting for the largest proportion (66.2%, 84.3% females), followed by behavioural syndromes associated with physical factors (22.1%, 100% females), and behavioural, emotional and tic disorders (22.1%, 82.4% females). Other common diagnoses included anxiety and stress-related disorders (20.8%, 88% females), schizophrenia spectrum disorders (15.6%, 41.7% females), and personality disorders (9.1%, 71.4% females). Notably, schizophrenia spectrum disorders were the only diagnosis group in which the proportion of male patients exceeded that of females. The majority (57.1%) of patients were assigned one single diagnosis, whilst all other patients received multiple diagnoses, with one patient receiving as many as five. All diagnoses are listed in Table 2 along with the main clinical characteristics of the study. The severity of illness (CGI-S), at baseline was in most patients classified as markedly ill (50.8%) or severely ill (32.5%), with an average CGI-S score of 5,4 ± 0,8 as listed in Table 2. 36 patients solely received quetiapine IR and 26 patients quetiapine XR, whilst 15 patients switched between the two formulations during the study period. In addition to quetiapine, two-thirds of the patients (66.2%) received one or more concomitant psychotropic medication at least once throughout the study, with 19.5% of all patients receiving fluoxetine (*n* = 15), 13.0% escitalopram (*n* = 10), 10.4% sertraline (*n* = 8), 6.5% mirtazapine (*n* = 5), and 6.5% aripiprazole (*n* = 5).

**Table 1:** Patient Characteristics

Number of patients, n	77
Number of assessments, n	327
Age (years), mean ± SD, range	15.06 ± 1.07 (12–17)
Gender, n (%) Male Female	17 (22.1) 60 (77.9)
Weight (kg), *n* = 73, mean ± SD, range	59.31 ± 12.83 (32.2–92.3)
BMI (kg/m²), *n* = 73, mean ± SD, range	21.3 ± 3.8 (14.3–31.4)
BMI Percentile, *n* (%) Normal- or underweight (P0–90) Overweight (> P90-97 Obese (≥ 97)	57 4 10

**Table 2:** Clinical Characteristics

Clinical centre, n (%)	
Bad Wildungen Parkland Klinik	6 (7.8)
Berlin Charité University Hospital	6 (7.8)
Freiburg University Hospital	2 (2.6)
Fulda Herz-Jesu-Hspital	2 (2.6)
Heidelberg University Hosital	4 (5.2)
Mannheim University Hospital	1 (1.3)
Munich LMU University Hospital	1 (1.3)
Tuebingen University Hospital	4 (5.2)
Ulm University Hospital	10 (13.0)
Vienna University Hospital	4 (5.2)
Wuerzburg University Hospital	28 (36.4)
Zurich University Hospital	9 (11.7)
Time from first to last assessment (days), mean ± SD, range	128.4 ± 107.1 (11–427)
Diagnosis, n = 77, n (%)	
F30 – F39 affective disorders	51 (66.2)
F50 – F59 behavioural syndromes associated with physical factors	17 (22.1)
F90 – F98 behavioural, emotional and tic disorders	17 (22.1)
F40 – F48 anxiety and stress-related disorders	16 (20.8)
F20 – F29 schizophrenia spectrum disorders	12 (15.6)
F60 – F69 personality disorders	7 (9.1)
F80 – F89 developmental disorders	3 (3.9)
F00 – F09 mental disorders due to physiological conditions	1 (1.3)
F10 – F19 mental disorders due to psychoactive substance use	1 (1.3
Severity of illness (CGI-S) *n* = 77 patients, n (%)	
Not at all ill	0
Borderline mentally ill	0
Mildly ill	0
Moderately ill	6 (7.8)
Markedly ill	39 (50.6)
Severely ill	25 (32.5)
Extremely ill	7 (9.1)
Co-medications n = 77, n (%)	
None	25 (32.5)
One co-medication	39 (50.6)
More than one co-medication	13 (16.9)

**Table 3:** Quetiapine Treatment Characteristics

Drug formulation,*n* (%)	
Immediate release	36 (46.8)
Extended release	26 (33.8)
Both	15 (19.5)
Dose (mg/d), n = 245 assessments, mean ± SD, range	276.8 ± 196.8 (25.0–800.0)
Weight-adjusted dose (mg/(kg x d)), n = 199 assessments, mean ± SD, range	4.5 ± 3.3 (0.3–13.7)
Serum concentration (ng/ml), n = 143 assessments, mean ± SD, range	125.8 ± 135.0 (0.0–678.0)

**Table 4:** Clinical Outcome

Therapeutic response, n = 218 assessments, n (%)	
Very good	40 (18.3)
Moderate	82 (37.6)
Weak	83 (38.1)
No response	13 (6.0)
Improvement of illness (CGI-I) n = 77 patients, n (%)	
Very much improved	8 (10.4)
Much improved	27 (35.1)
Minimally improved	25 (32.5)
No change	8 (10.4)
Minimally worse	6 (7.8)
Much worse	3 (3.9)
Very much worse	0
Adverse drug reactions n = 72 patients, n (%)	
No ADRs	19 (26.4)
ADRs not significantly interfering with patient functioning	42 (58.3)
ADRs significantly interfering with patient functioning	11 (15.3)
ADRs outweighing the therapeutic effect	0

### Serum concentration in relation to daily dose and weight-adjusted dose

The average daily quetiapine dose in the study sample was 276.8 ± 196.8 mg and ranged from 25.0 mg to 800.0 mg, with only five patients receiving doses higher than 600 mg/d. The average mean daily dose per kg body weight was 4.5 ± 3.3 mg/kg, ranging from 0.3 mg/kg to 13.7 mg/kg. Boys received significantly higher absolute mean daily doses of 401.2 ± 188.8 mg, as well as weight-adjusted mean daily doses of 5.8 ± 2.9 mg/kg than girls with a mean daily dose of 235.6 ± 181.9 mg (*p <* 0.001) and an average weight-adjusted daily dose of 4.6 ± 3.3 mg/kg (*p* < 0.001). Patients diagnosed with schizophrenia spectrum disorder were prescribed the highest mean daily dose (562.5 ± 144.8 mg), as well as the highest weight-adjusted daily dose (8.6 ± 2.8 mg/kg), whilst patients suffering from behavioural syndromes associated with physical factors received the lowest mean daily dose (132.4 ± 98.7 mg) and the lowest weight-adjusted daily dose (2.5 ± 2.1). In general, there was a significant difference between the mean daily doses in the different diagnostic groups (X² = 31.513, *p* < 0.001), as well as between the weight-adjusted daily doses (X² = 29.007, *p* < 0.001) (Table 3, 4).

In total, 143 quetiapine serum concentrations referring to 70 patients were available. The mean serum concentration was 125.8 ± 135.0 ng/ml and ranged from 0.0 ng/ml to 678.0 ng/ml. The mean serum concentration measured in boys (152.4 ± 105.2 ng/ml) was significantly higher than the serum concentration measured in girls (115.1 ± 144.4 ng/ml, *p* < 0.001). The highest serum concentration was measured in patients diagnosed with schizophrenia spectrum disorders (254.5 ± 209.7 ng/ml), whilst the lowest serum concentration was measured in patients diagnosed with behavioural syndromes associated with physical factors (82.3 ± 117.7 ng/ml). However, there was no significant difference in concentrations between the different diagnostic groups (X² = 11.610, *p* < 0.071), and likewise no significant difference between the comedications fluoxetine, escitalopram, sertraline, mirtazapine, aripiprazole and no comedication was observed (X² = 11.095, *p* < 0.050). However, the concentration difference between patients taking quetiapine IR (100.0 ± 122.6 ng/ml) and XR (158.6 ± 146.1 ng/ml) was substantial, *p* < 0.001.

A positive mean correlation was observed between the absolute daily dose and the serum concentration (*r*_s_ = 0.661, *p* < 0.001) as well as between the weight- adjusted daily dose and the serum concentration (*r*_s_ = 0.580, *p* < 0.001). The variation in the absolute and the weight- adjusted daily dose explained 31.9% (*r*_s_ ² = 0,319, *p* < 0.001) and 26.5% (*r*_s_ ² = 0.265, *p* < 0.001) of the variability in serum concentrations respectively. A multiple linear regression was calculated to predict the serum concentration based on the dose, weight, gender, and the presence of concomitant medications. These variables statistically significantly predicted the serum concentration, explaining 34.9% of the variability in serum concentration (*r*_s_ ² = 0.349, *p* < 0.001). The dose-related concentration (DRC) factor calculated for quetiapine was 0.6 ± 1.0. The drug formulation showed no significant influence on the DRC-factor with a mean DRC-factor of 0.6 ± 1.3 for quetiapine IR and of 0.5 ± 1.3 for quetiapine XR (*p* = 0.063). In addition, the patients showed no significant difference in DRC-factors depending on the concomitant medications taken (*p* = 0.849).

### Clinical effectiveness

The clinical effectiveness of medication rated from 1 (very good) to 4 (no response) was available for 93.5% (*n* = 72) of all patients with a mean score of 2.3 ± 0.8. In 18.4% (*n* = 40) of the assessments, the clinical response to quetiapine was rated as ‘very good’ and in 37.6% (*n* = 82) as ‘moderate’, meaning that a positive response to quetiapine was reported in 56.0% (*n* = 122) of all 218 assessments. A ‘weak’ therapeutic response was documented in 38.1% (*n* = 83) of the assessments and ‘no response’ in 6.0% (*n* = 13). The best clinical response during the final assessment was recorded in patients suffering from different kinds of schizophrenia spectrum disorders (2.0 ± 1.0). Patients suffering from paranoid schizophrenia (2.2 ± 0.8) showed very good response. Likewise, patients suffering from severe depressive episodes with psychotic symptoms (1.8 ± 1.0) also showed very good response. The lowest efficacy was recorded in patients diagnosed with behavioural, emotional and tic disorders (2.7 ± 0.8) and in patients with anxiety and stress related disorders (2.7 ± 0.6). Despite the different mean values, the statistical difference of the effectiveness between the diagnoses was not significant (X² = 8,455, *p* = 0,207).

The severity of illness measured according to the CGI-S score had improved from 5.4 ± 0.8 at baseline to 4.5 ± 1.2 during the last assessment. However, no significant relationship between the severity of illness and the therapeutic response was found (*r*_s_ = 0.199, *p* = 0.094). Likewise, the clinical effectiveness was not influenced by the patients’ age (*r*_s_ = 0.018, *p* = 0.791).

A significant relationship was observed between the weight- adjusted daily quetiapine dose and the therapeutic response (*r*_s_ = 0.161, *p* = 0.031), as well as between the quetiapine serum concentration and the therapeutic response (*r*_s_ = 0.238, *p* = 0.006).

Information based on the CGI-I was available for all 77 patients. The mean CGI-I score of all patients was 2.8 ± 1.2. 10.4% (*n* = 8) of the patients’ improvement was rated as ‘very much improved’, 35.1% (*n* = 27) as ‘much improved’ and 32.5% (*n* = 25) as ‘moderately improved’, 10.4% (*n* = 8) of all patients experienced ‘no change’, whilst 7.8% (*n* = 6) of all patients’ conditions became ‘moderately worse’, 3.9% (*n* = 3) became ‘much worse’, and no patients’ condition became ‘very much worse’. The symptoms documented on the Pediatric Adverse Events Rating Scale that had improved most during treatment with quetiapine between baseline and final assessment were suicidal thoughts (−55.8%), angry mood (−44.4%), reduced appetite (−42.1%), suicidal behaviour (−36.4%), affective flattening (−28.6%), and mood swings (−21.9%).

### Adverse drug reactions

84.7% (*n* = 61 patients) of the 77 patients with information given regarding ADRs, experienced no ADRs or ADRs which were not significantly interfering with the patients’ functioning. No patient experienced ADRs outweighing the therapeutic effect. However, 15.3% (*n* = 11) of the patients experienced ADRs significantly interfering with patient functioning at one or more times during study. In disregard of the most frequent ADR weight gain, exhaustion, increased appetite, and somnolence were the most common ADRs reported in 30.7%, 18.7% and 12.0% of the patients respectively. Other common ADRs were dry mouth, tachycardia, dizziness and headache each evident in 8.0% of the patients. After adjusting all patients’ weight to age adjusted weight percentiles (Neuhauser et al. 2013), 60.27% of all patients had experienced weight gain in the course of the study. After a mean of 128.4 ± 107.1 days, the mean weight of all 73 Patients had increased by 3.2 ± 4.8 kg. After converting all values into percentiles (Neuhauser et al. 2013), the mean percentile had increased by 6.0 ± 11.4%. Neither the daily dose (*r*_s_ = 0.141, *p* = 0.342), nor the serum concentration (*r*_s_ = 0.132, *p* = 0.133) showed a significant impact on the occurrence of side effects.

### Estimation of preliminary therapeutic reference ranges for children and adolescents

The comparison of the serum concentrations in our present sample with the recommended TRR defined for adult patients with schizophrenia of 100–500 ng/ml (Hart et al. 2026) revealed that 38.5% (*n* = 50) of all measured serum concentrations (*n* = 130) were within the recommended range, whereas 58.5% (*n* = 76) of the serum concentrations were below and only 3.1% (*n* = 4) were above. In minors with schizophrenia spectrum disorder (*n* = 28), 67.9% (*n* = 19) of all serum concentrations were within the recommended TRR and only 21.4% (*n* = 6) below and 10.7% (*n* = 3) above. According to the method by the AGNP expert group of TDM in Psychiatry (Hart et al. 2026) as described above, a rough estimation of a TRR was determined by measuring the range between the arithmetic mean ± one standard deviation of the serum concentrations in responding patients. The arithmetic mean ± standard deviation of all quetiapine serum concentrations of responders regardless of their diagnosis (*n* = 62) was 152.3 ± 138.4 ng/ml, which leads to a preliminary therapeutic reference range with a lower limit of about 14 ng/ml and an upper limit of about 291 ng/ml. Based on the values of patients with affective disorders (*n* = 38), a TRR between about 5 ng/ml and 247 ng/ml was calculated; whereas for responding patients with schizophrenia spectrum disorder (*n* = 19) a range between about 59 ng/ml and 380 ng/ml was obtained. Based only on a small number of assessments, for adolescents with anxiety and stress-related disorders (*n* = 19), a TRR between about 19 ng/ml and 286 ng/ml was found. A TRR between 0 ng/ml and 164 ng/ml was suggested for patients diagnosed with behavioural syndromes associated with physical factors (*n* = 7) and a TRR between 9 ng/ml and 234 ng/ml for patients with behavioural, emotional and tic disorders (*n* = 8).

In addition, preliminary TRRs were calculated according to the 25th and 75th percentiles of patients defined as responders to quetiapine treatment. Based on this method, the preliminary transdiagnostic TRR suggested was about 53–234 ng/ml. A preliminary TRR of 38–205 ng/ml was calculated for patients suffering from affective disorders and for patients diagnosed with schizophrenia spectrum disorders a TRR of 115–308 ng/ml was proposed. For patients with anxiety and stress-related disorders a preliminary TRR of 65–290 ng/ml was calculated, for patients with behavioural syndromes associated with physical factors a TRR of 27–83 ng/ml, and for patients with behavioural, emotional and tic disorders a TRR of 25–250 ng/ml. All diagnose-specific preliminary reference ranges, based on both calculation methods, are presented in Table 5.

**Table 5:** Preliminary therapeutic reference ranges subdivided into different diagnoses, calculated by mean ± SD, and by 25th and 75th percentiles. I

Diagnosis	Serum concentrations, *n*	Serum concentrations of responders, *n* (%)	Serum concentration of responders (ng/ml) mean ± SD	Therapeutic reference range (ng/ml) derived from mean ± SD	Therapeutic reference range (ng/ml) derived from 25th – 75th Percentiles
F20-29 schizophrenia spectrum disorders	28	19 (68%)	219.3 ± 160.7	59–380	115–308
F30-39 affective disorders	91	38 (42%)	126.1 ± 120.7	5–247	38–205
F40-48 anxiety and stress-related disorders	24	6 (25%)	152.3 ± 133.4	19–286	65–290
F50-59 behavioural syndromes associated with physical factors	23	7 (30%)	81.3 ± 82.4	0–164	27–83
F90-98 behavioural, emotional and tic disorders	19	8 (44%)	121.8 ± 112.6	9–234	25–250
All diagnoses	130	62 (48%)	152.3 ± 138.4	14–291	53–234

Including number of serum concentrations of all patients and of patients defined as responders to quetiapine, as well as serum concentrations (ng/ml)

*SD: standard deviation*

## Discussion

### Relationship between daily dose and serum concentration

Consistent with extended literature for adults (DeVane and Nemeroff 2001; Sparshatt et al. 2011), as well as for children and adolescents (Albantakis et al. 2017; Gerlach et al. 2007), a positive linear relationship between the absolute- as well as between the weight-adjusted daily dose and the serum concentration of quetiapine was observed. A high inter-patient variability in serum concentrations was observed with variation in weight-adjusted daily dose explaining only 26.5% of the variability in serum concentration. Available literature describes similar high inter-individual variability for second generation antipsychotics in paediatric patients (Albantakis et al. 2017; Egberts et al. 2020; Taurines et al. 2022), supporting the indication for routine TDM when treating children and adolescents. Elsewhere, it was even shown that there is no significant correlation between the daily dose and the serum concentration of quetiapine (Sachse et al. 2003). Such differences in inter-individual serum levels as well as other altered drug responses could be due to individual differences in renal- or hepatic function, weight, volume of distribution or genetic polymorphism of metabolizing enzymes. Quetiapine is predominantly metabolized via CYP3A4. CYP3A4 activity varies significantly with age, with newborns and infants enzyme activity generally being lower than in adults. However, children between the ages of 1 and 9 years can in some cases display higher CYP3A4 activity than adults, potentially resulting in faster drug metabolism and altered dosing needs (Lu and Rosenbaum 2014). As children grow older, CYP3A4 activity gradually increases, typically reaching adult levels by adolescence. Concomitant medication that strongly modulates the activity or expression of CYP3A4 could possibly lead to changing serum concentrations of quetiapine (Grimm et al. 2006). In literature, interactions between quetiapine and escitalopram have been described (Castberg et al. 2007). However, the concentration-dose relationship in our study was not significantly influenced by any of the five examined concomitant medications fluoxetine, escitalopram, sertraline, mirtazapine or aripiprazole, despite CYP3A4 being involved in the metabolization of aripiprazole, escitalopram and mirtazapine (Hart et al. 2026), and a potential risk of pharmacokinetic interactions of escitalopram when combined with quetiapine having been described in literature (Castberg et al. 2007).

Generally, age is known to influence the pharmacokinetics of second generation antipsychotics possibly due to decreasing hepatic metabolism and -renal glomerular filtration (Aichhorn et al. 2006). This often leads to higher serum concentrations of antipsychotics in elderly patients (Aichhorn et al. 2005; Weiss et al. 2005). Castberg et al. (2007) reported that patients aged between 14 and 17 years showed lower dose related serum concentration levels of quetiapine than adults aged between 18 and 70 years and that patients above the age of 70 years had even higher concentrations, suggesting that higher doses than usual should be considered when treating minors. Likewise Aichhorn et al. (2006) showed significantly higher DRC-factors with age, resulting in an average increase of 11% per 10 years. In our study, the mean DRC-factor for quetiapine IR was 0.6 ± 1.3, which was only slightly higher than the value reported in the 2018 consensus guidelines for adults of 0.54 (0.31–0.78) (Hiemke et al. 2018) and the updated consensus guideline by Hart et al. (2026) of 0.55 (0.42–0.82). Fekete et al. (2020) had reported a considerably lower mean DRC-factor for quetiapine IR in minors aged between 10 and 17 years of 0.30 (0.13–0.48). The reason for our comparatively high DRC-factor lies partly in one specifically high serum concentration measured with a DRC factor of 10.5. Since only 74 serum concentrations were included in the calculation, this one DRC-factor led to a substantial distortion of the mean result. However, according to expectation, the mean DRC-factor calculated for quetiapine XR in our study of 0.5 ± 1.3 was considerably lower than the DRC-factor described in the 2018 consensus guideline for adults of 0.97 (0.29–1.66) (Hiemke et al. 2018). The updated consensus guidelines by Hart et al. (2026) describe the highest DRC-factor of 1.074 (0.933–1.264). The most likely explanation for this discrepancy is the different timing of blood sampling. In the consensus guidelines the serum concentration was determined 10 h after drug intake rather than at trough level, which is expected to result in higher measured concentrations and, consequently, higher DRC factors.

The quetiapine formulation did not have a significant impact on the DRC-factor in our study. This observation is consistent with results from former studies which observed similar AUC values and apparent oral clearance for both formulations, observing that when administered at the same daily dose quetiapine XR and quetiapine IR have comparable bioavailability at steady state (Figueroa et al. 2009). In contrast, Albantakis et al. (2017) had observed lower mean serum concentrations in patients treated with quetiapine IR compared to patients treated with quetiapine XR. According to previous studies examining pharmacokinetic differences between quetiapine IR and XR (Figueroa et al. 2009; Mamo et al. 2008), the trough concentrations for both formulations were similar, which is why these higher concentrations measured with quetiapine XR could possibly be the result of incorrect dating of blood sampling. Even though sampling at trough was requested, in practice the time of blood withdrawal is often normalised to the convenience of the physician and patient.

### Therapeutic response

Although not comparable with previous clinical efficacy studies due to different terms of methodology, our study provided corresponding results on therapeutic response with former studies in adults (Buckley 2004; Riedel et al. 2011), as 56.0% of children and adolescents had experienced a ‘very good’ or ‘moderate’ response. This result refers to symptoms from all types of mental, behavioural, and neurodevelopmental disorders whilst most comparable studies concentrated on diagnosis-specific assessments of efficacy. Despite the best response being observed in patients suffering from schizophrenia spectrum disorder, no significant difference in therapeutic response was documented between the individual diagnoses or diagnostic groups in our study, underlining evidence of the broad-based effectiveness of quetiapine. This effect could have been influenced by the small study population and the small number of patients within each of the diagnoses. The proportion of patients with symptoms rated as ‘much improved’ or ‘very much improved’ on the CGI-I scale was 45.6%. This result seems rather modest considering the fact that Findling et al. (2012) documented percentages of approximately 60% for patients diagnosed with schizophrenia of a similar age, and Shaw et al. (2001) documented considerably higher percentages of up to 93% for patients mainly diagnosed with schizophrenia in the same age group. However, Shaw et al. had used a very small study sample of only 15 patients, reducing the reliability of the study results. Our comparably low percentage may be due to the markedly lower mean daily dose in our study of 276.8 ± 196.8 mg/d compared to mean daily doses of up to 800 mg/d in the two studies cited, seeing as in adult patients quetiapine has shown better efficacy at higher doses, especially in the treatment of positive schizophrenia symptoms, than at lower doses (Buckley 2004). Potentially dose-dependent onset of symptom improvement has been discussed in former studies for adults and adolescents (Findling et al. 2012; Tandon 2003) and in line with this, a correlation between the dose and the therapeutic response was observed in our study. Yet studies have demonstrated that the mean dose leading to the best possible efficacy varies strongly depending on the diagnosis (Zhornitsky et al. 2011). Interestingly, despite the correlation found, patients showing ‘no therapeutic response’ had received nearly the same mean daily dose as patients with ‘very good’ therapeutic response. Those patients not responding seemed to show poor or no response at all, irrespective of the prescribed dose. The symptom that improved by far the most was ‘suicidal thoughts’, which is consistent to results from previous studies stating that although both positive and negative symptoms tend to decrease, the change of symptoms caused by second generation antipsychotics is generally more pronounced for positive symptoms (Shaw et al. 2001). Unlike the results in our study, Albantakis et al. (2017) observed no correlation between the quetiapine serum concentration and the therapeutic effects in minors. As discussed by Albantakis et al., one reason for missing correlation could be due to non-responders not being eliminated from the study though this was not the case in our study either. They also argument that correlation could be missing because they only evaluated the last available valid TDM assessment per patient, not considering the treatment response at the beginning. In our study, however, the assessment of clinical response was assessed at each time point of assessment and in case of several concomitant medications for every medication separately, in order to enhance the reliability of the results. No significant relationship was observed between the severity of illness and therapeutic response. However, this finding should be interpreted with caution, as the heterogeneous study population included patients with a wide range of psychiatric diagnoses, which may have obscured diagnosis-specific associations. Moreover, the relatively limited sample size within individual diagnostic groups reduced the statistical power to detect potential differences. To date, evidence regarding the relationship between illness severity and therapeutic response to quetiapine remains scarce. Similar to our findings, Datto et al. (2016) reported that although patients with bipolar I disorder exhibited a faster initial treatment response than those with bipolar II disorder, both groups achieved comparable symptom improvement after 8 weeks of treatment, suggesting that baseline illness severity did not substantially influence the overall therapeutic outcome.

### Adverse drug reactions

Although ADRs were very common and documented in 73.6% of all children and adolescents treated with quetiapine, the majority of patients experienced only mild ADRs not significantly interfering with patient functioning, in line with former reports in children and adolescents (Findling et al. 2012; Joshi et al. 2012). Severe ADRs significantly interfering with patient functioning were only seen in 8 patients (11.1%). This finding is in line with comparable adult studies (Buckley 2004; Riedel et al. 2007), suggesting that quetiapine appears to be relatively well tolerated also in this age group. Generally the adverse event profile of quetiapine observed in former studies in minors consists of the most common adverse events being somnolence, headache, dizziness, insomnia and gastrointestinal disturbances (Albantakis et al. 2017; Delbello et al. 2006; Findling et al. 2012; Joshi et al. 2012; Pathak et al. 2013; Shaw et al. 2001), which is greatly consistent with ADRs documented in our study.

Indications for potential dose-dependent occurrence of ADRs has been displaced, with increased incidence of ADRs at higher doses (Murasaki et al. 2018). However, in our study no significant relationship was found between the dose and the occurrence of ADRs in accordance with most other findings in literature (Findling et al. 2012; Pathak et al. 2013). Additionally, in line with former studies in minors (Albantakis et al. 2017; Gerlach et al. 2007), no significant correlation was observed between the serum concentration and the occurrence of ADRs either. This indicates that certain patients seem to be more vulnerable to side effects regardless of the concentration. Interestingly, better therapeutic response was mostly observed in patients with greater vulnerability to ADRs, whilst lower response was often associated with less ADRs. This result emphasizes the high inter-patient variability, supporting the indication for TDM in children and adolescents.

Even though second generation antipsychotics are known to have a significantly diminished risk of inducing extrapyramidal symptoms, an increased risk for weight gain in second generation antipsychotics has been discussed (Leucht et al. 2009). Furthermore, there are suggestions that children are more sensitive to metabolic adverse events than adults, indicating that children have shown proportionately greater and more rapid weight gain compared to adults (Correll and Carlson 2006; Vitiello et al. 2009). The average weight gain observed in our study was 3.2 ± 4.8 kg over a mean period of 128.4 ± 107.1 days. Because of the natural propensity for adolescents to gain weight, the weight was converted into age dependent percentiles resulting in an average percentile gain of + 6.0 ± 11.4%. Generally, considering the shorter observation period than in most other comparable studies (Findling et al. 2012; Joshi et al. 2012), the average weight gain observed in our study was slightly lower than in other studies in children and adolescents (Baeza et al. 2017; Correll et al. 2009), more likely comparable to results of adult studies (Murasaki et al. 2018; Pérez-Iglesias et al. 2014), suggesting further investigation on age dependent weight change due to treatment with quetiapine.

### Suggestion of a therapeutic reference range

For adults with schizophrenia a therapeutic reference range for quetiapine of 100–500 ng/ml has been defined (Hart et al. 2026; Hiemke et al. 2018). No comparable reference range has been described for children and adolescents treated with quetiapine, though generally similar pharmacokinetic profiles of quetiapine in minors compared to profiles in adults have been observed (McConville et al. 2000; Winter et al. 2008). The comparison of concentrations in our paediatric sample with the established therapeutic reference range for adults revealed that only 38.5% of all serum concentrations were within the recommended range, whereas 58.5% of the serum levels were below. For patients diagnosed with a schizophrenia spectrum disorder, 67.9% of all serum concentrations were within the recommended range, and only 21,4% were below. Since the majority of patients in our study responded well to quetiapine, our results suggest that the recommended range for minors diagnosed with schizophrenia, and even more for minors using quetiapine for other psychiatric diagnoses such as anxiety disorders or depressions, could be considerably lower than the range defined for adults diagnosed with schizophrenia. Similar results have been reported by Albantakis et al. (2017), with 67.8% of minors showing a serum concentration below the suggested reference range defined for adults.

In order to define a reference range for minors, we referred to a method described by the consensus guidelines for TDM in neuropsychopharmacology (Hart et al. 2026; Hiemke et al. 2018) by using the arithmetic mean ± one standard deviation of the serum concentrations in the blood of responders to estimate the upper and lower limit. This method does not take the occurrence of ADRs into account, which in our case however is not of great relevance, as no relationship between the serum concentration and the occurrence of ADRs was observed. Referring to this method, a therapeutic reference range for minors of 14–291 ng/ml, considerably lower than the recommended range for adults, was calculated. As the therapeutic reference range for adults is defined for patients with schizophrenia, we also calculated a reference range for minors diagnosed with schizophrenia spectrum disorder of 59–380 ng/ml, suggesting that patients suffering from schizophrenia need to obtain higher serum concentrations in order to respond to quetiapine than patients suffering from most other psychotic disorders. Accordingly, an estimated reference range was calculated for minors suffering from affective disorders, resulting in a reference range of 5–247 ng/ml, similar to the transdiagnostic range calculated for minors. For the sake of completeness, a therapeutic reference range was also suggested for all other diagnoses with responding assessments ≥ 5. However, due to the very small number of assessments, the results of those three diagnoses should be interpreted highly critically. The additional reference ranges calculated according to the 25th and 75th percentiles, displayed smaller serum concentration intervals for most diagnoses. Moreover, the reference range proposed by the 25th and 75th percentiles for patients suffering from behavioural syndromes associated with physical factors of 27–83 ng/ml is presumably more precise than the reference range calculated by the mean ± SD of 0–164 ng/ml, given that a therapeutic response at 0 ng/ml seems highly unlikely. Consequently, this method should definitely be considered for further studies on therapeutic reference ranges. To summarize, our findings suggest therapeutic reference ranges significantly lower than the range described for adults. Especially the calculated lower limits deviate strongly, suggesting further prospective and large controlled studies with fixed dose-design as well as observational studies to verify or correct the defined reference ranges and to obtain further results regarding other indications for the use of quetiapine in minors.

### Limitations

Naturalistic designs, as seen in our study, allow an assessment close to real-life situations, reflecting the prescription patterns in a population more accurately than controlled studies. The naturalistic conditions were mainly achieved through little entry requirements, including patients with multiple different psychiatric diagnoses and target symptoms, comorbid conditions, and concomitant medications. Nevertheless, results should be interpreted carefully, due to several methodological limitations. For one, data from naturalistic studies are vulnerable to bias which in the present study could have been caused by variable psychiatrists evaluating the conditions or variable dosing regimens, by the influence of co-medications, by variable histories of treatment with psychotic medications, or by doubtful adherence. Moreover, the sample size in our study was limited, and certain subgroups were often too small and heterogenous to identify variables with small effect size. Furthermore, within naturalistic studies, the relationship between serum concentration and therapeutic response or adverse events is difficult to determine, seeing as patients who respond to placebo and non-responders, who are likely to receive higher dosages, nor patients with ADRs, likely to receive lower dosages, are not excluded from the study. In our study, for most evaluations, all valid TDM assessments were included in the analysis, enabling the assessment of the treatment response at different times throughout the study and increasing the sample size. However, this also means that certain patients were included more often in the evaluation of sub-analyses than others, depending on the number of valid TDM assessments, potentially leading to inaccurate results due to inter-patient variability of response. Intraindividual longitudinal analyses would have been interesting to monitor the development, seeing as factors such as weight change, adverse drug reactions and efficacy can change over time. This type of analysis was not performed in this study seeing as only a small sample size of 19 patients had taken the medication for longer than 6 months which would have not allowed for statistically meaningful interpretation of the data. The CGI-rating scale used to assess improvement can also be seen critically, because only one overall score is determined for each patient, even though patients have been diagnosed with several different diagnoses and treated for different target symptoms, so that it is unclear which diagnosis the score refers to. The therapeutic response however was rated separately for each diagnosis, providing more accurate information on the effect of quetiapine. All limitations discussed above may have significant influence on the results of our study and should be considered when applying any of the results to clinical practice.

## Conclusion

Our present study demonstrated a linear relationship between the daily dose and the serum concentration of quetiapine in children and adolescents despite high inter-patient variability. Generally, quetiapine has shown to be a well-tolerated antipsychotic with good clinical response in children and adolescents, comparable to results observed in adults. Therapeutic response, unlike adverse events, was significantly influenced by the daily dose and most importantly by the serum concentration. Our results suggest a lower therapeutic reference range for children and adolescents with schizophrenia and other diagnoses compared to the recommended reference range described for adults with schizophrenia. However, to verify and strengthen our preliminary reference ranges, further data should be maintained through controlled studies with larger sample sizes. This naturalistic study has significantly increased the amount of available data for quetiapine in minors, emphasising the vulnerability of this population and hence supporting the importance for routine TDM.

**Fig. 1:**
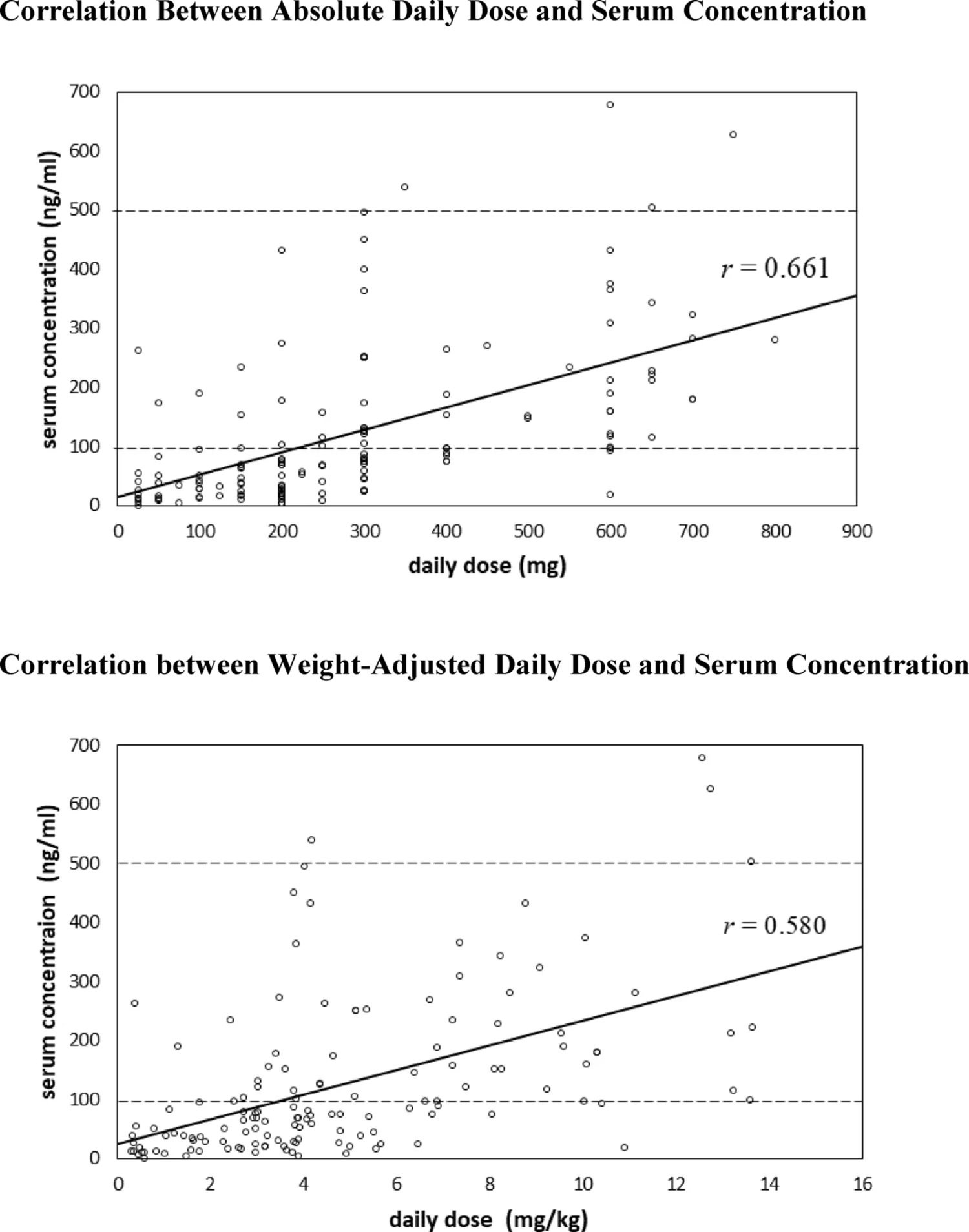
This figure illustrates the positive linear relationship between both absolute daily dose (*r*_s_ = 0.661, *p* < 0.001) and weight-adjusted daily dose (*r*_s_ = 0.580, *p* < 0.001) with serum concentrations (*n* = 143 assessments from 70 patients). The upper and lower limit of the recommended therapeutic reference range for adults of 100–500 ng/ml is indicated by two dotted lines

**Fig. 2:**
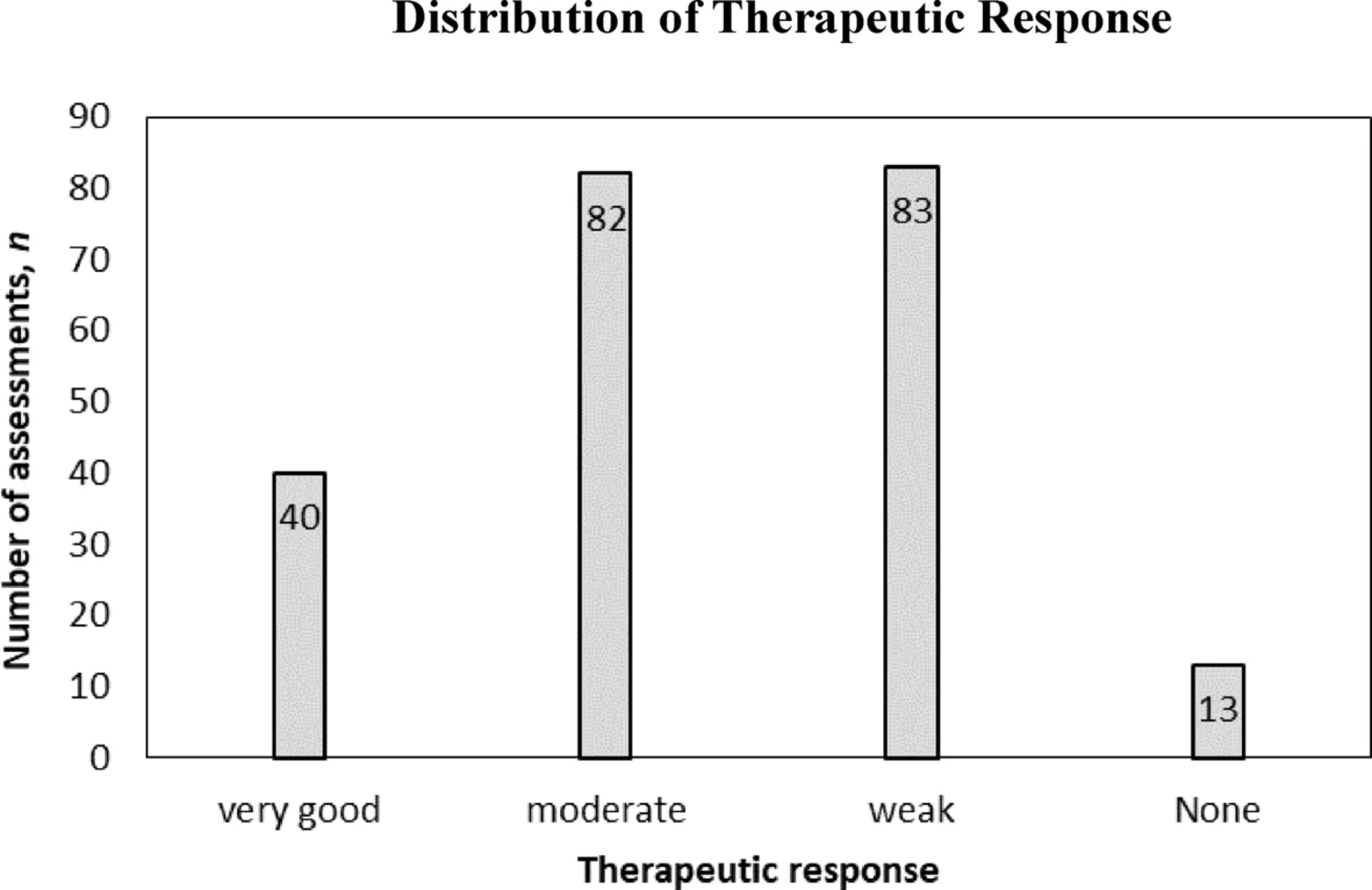
A visualisation of the clinical response scores (1=very good to 4=no response) for all 218 assessments after baseline (n = 72 patients), showing that 56.0% of patients were classified as responders

**Fig. 3:**
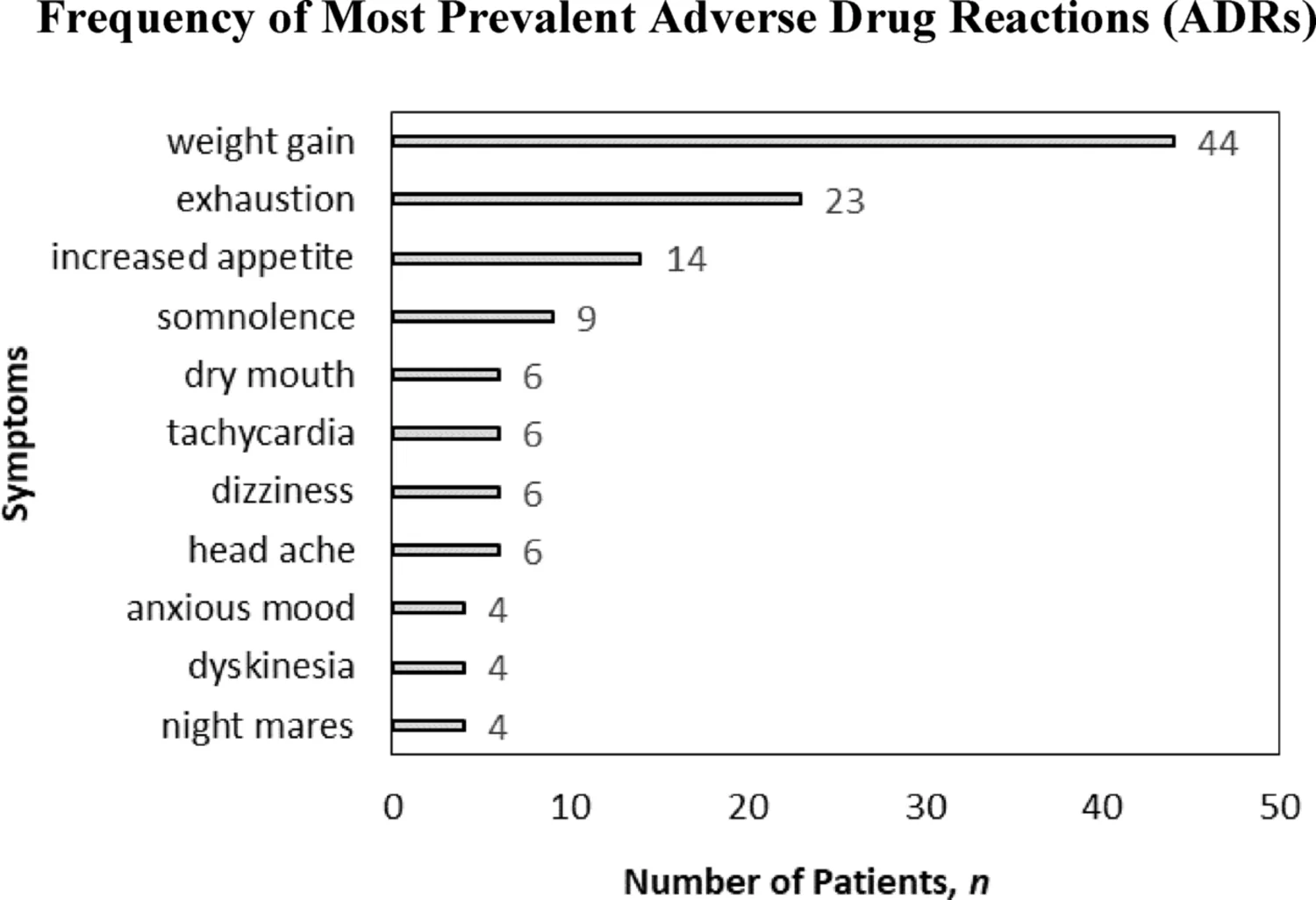
This chart illustrates the most frequent ADRs observed during at least one visit throughout the study, within the population of n = 75 patients. The most frequent ADRs shown are weight gain (58.7%), exhaustion (30.7%), and increased appetite (18.7%)

**Fig. 4:**
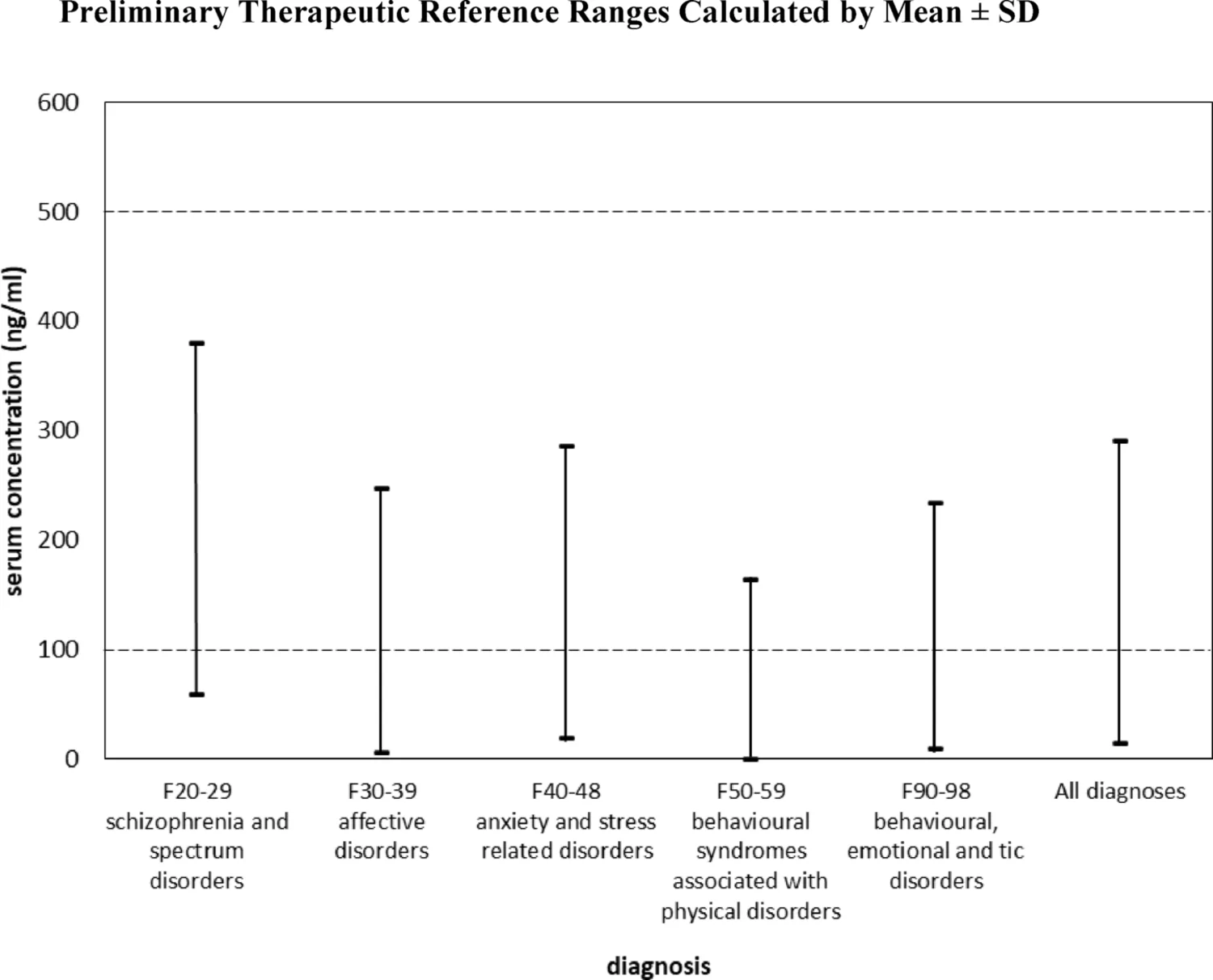
The figure illustrates the estimated therapeutic reference ranges for the following diagnostic groups calculated by 25th and 75th percentiles: schizophrenia spectrum disorders (F20-29), affective disorders (F30-39), anxiety and stress related disorders (F40-48), behavioural syndromes associated with physical factors (F50-59) and behavioural, emotional and tic disorders (F90-98). The dotted horizontal lines indicate the recommended therapeutic reference range for adults of (100–500 ng/ml)

**Fig. 5:**
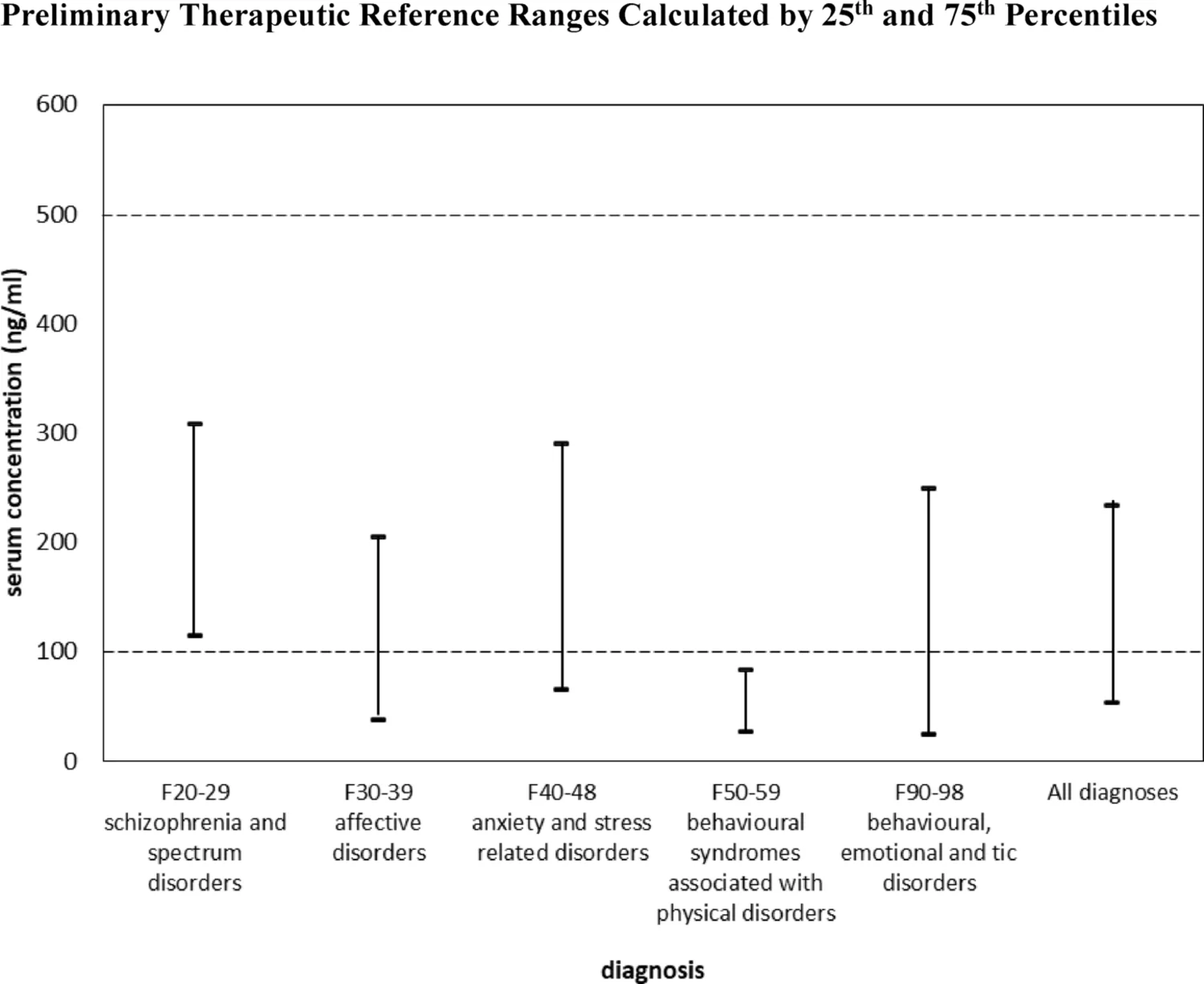
The figure illustrates the estimated therapeutic reference ranges for the following diagnostic groups calculated by mean ± SD: schizophrenia spectrum disorders (F20-29), affective disorders (F30-39), anxiety and stress related disorders (F40-48), behavioural syndromes associated with physical factors (F50-59) and behavioural, emotional and tic disorders (F90-98). The dotted horizontal lines indicate the recommended therapeutic reference range for adults of (100–500 ng/ml)

## Supplementary Information

Below is the link to the electronic supplementary material.

Supplementary Material 1

## Data Availability

No datasets were generated or analysed during the current study.
