# Supplementary material for: Therapeutic drug monitoring in children and adolescents treated with quetiapine for different psychiatric disorders: results of the TDM-VIGIL pharmacovigilance study
Source: J Neural Transm (Vienna). 2026 Aug 17;133(9):2715–31. doi: 10.1007/s00702-026-03253-6 (PMC13687216; doi:10.1007/s00702-026-03253-6)
Supplement: Supplementary file 1 [file 702_2026_3253_MOESM1_ESM.docx]

Dear editors,

please find the enclosed manuscript "Therapeutic drug monitoring in children and adolescents treated with quetiapine for different psychiatric disorders: results of the TDM-VIGIL pharmacovigilance study". We hope you find it appealing and will consider it for publication in the “Journal of Neural Transmission”. The manuscript is one of several publications resulting from TDM-VIGIL project, a prospective multicentre pharmacovigilance study funded by the German Federal Institute for Drugs and Medical Devices (BfArM). It was not submitted for publication elsewhere.

The study described in our manuscript aimed to investigate the relationship between dose and serum concentration of quetiapine and the factors influencing them in children and adolescents treated for various psychiatric disorders. Our results showed a significant correlation between quetiapine dose and serum concentrations. We also identified preliminary therapeutic reference ranges for minors with schizophrenia-spectrum disorders, affective disorders and behavioural/emotional/tic disorders.

We very much hope that the manuscript will be considered for publication. If you have any questions, please do not hesitate to contact us.

Thank you in advance for your consideration of this manuscript.

Kind regards

on behalf of the authors

Christian Fleischhaker
